# Comment on: Risk factors for workplace encounters with weapons by hospital employees^☆^

**DOI:** 10.1016/j.puhip.2022.100256

**Published:** 2022-04-07

**Authors:** Chidinma Okani, Carmen Black

**Affiliations:** Department of Psychiatry, Yale School of Medicine, United States; Department of Psychiatry, Yale School of Medicine, United States

Blando et al. [1] highlighted in their recent cross-sectional survey that “metal detectors are an important and effective intervention” due to their ability to assumedly reduce healthcare workplace violence. However, we write today as Black physicians with academic expertise in antiracism with several concerns regarding the paper's central premise, design, and conclusions.

We, the authors, value the ultimate goal of hospital security protocols to reduce the incidence and severity of healthcare violence. Nonetheless, this paper relies upon the premise that armed violence is a leading cause of hospital assaults and that reducing weapons entering hospital grounds through metal detectors successfully mitigates that risk. Although healthcare violence is distressingly common, Kelen characterized US hospital-based shootings from 2000 to 2011 [2], and concluded that less than 2% of all workplace shootings involved the healthcare sector. Indeed, they calculated a greater statistical likelihood of being struck and killed by lightning than being the victim of a hospital shooting.

Authors use the number of weapons confiscated by metal detectors as an inaccurate proxy for the ultimate desired outcome of any hospital safety campaign: effectual reductions in workplace violence. However, none of the sources cited within this paper lend measured evidence regarding the efficacy of metal detectors in reducing actual incidences of hospital violence, armed or otherwise. For example, the paper's introduction cites a 1999 study by Rankins [3] showing that the implementation of emergency department metal detectors doubled the number of weapons confiscated from patients, yet failed to significantly decrease the number of armed or unarmed assaults at all. Beyond this insignificant finding, no other cited study measures the end objective of reduced hospital assaults whatsoever. Moreover, even if metal detectors were in place, Kelen determined that the determined motives behind hospital shooters meant that less than half of shootings occurring inside a hospital might have been thwarted by metal detectors [2].

Authors cite numerous studies regarding the prevalence and consequences of general healthcare violence. However, the etiologies and solutions for unarmed/general hospital violence are not automatically generalizable to armed hospital violence. For example, authors cite a 2010 study by Roche [4] attempting to support the notion that weapons entering the hospital causes violence, and fear of violence amongst providersmight then cause medical mistakes. Instead of armed violence, Roche actually states that workplace violence was associated with clinical “unit operations” including “unanticipated changes in patient mix; proportion of patients’ awaiting placement; the discrepancy between nursing resources required form acuity measurement and those supplied; more tasks delayed, and increases in medication errors.” This suggests that poorly funded clinical care may increase the likelihood of the most common forms of hospital violence, being unarmed violence. Therefore, diverting money away from clinical operations toward dubiously effective, yet costly, policing measures for rare incidents of armed assault might actually be passively increasing versus decreasing hospital violence through clinical resource deprivation and frustrated patients.

Authors concede the limitations of recall and reporting bias, given that only 77 out of 2200 solicited security personnel completed the self-reported, cross-sectional survey. However, security personnel employed by hospitals with metal detectors are inherently biased towards seeing those policing practices as necessary and effective, given that their livelihoods depend on them. Furthermore, authors measured whether “perceived” high risk of violence and “responses” to violent communities were associated with weapons confiscation. From an antiracist perspective, “violence” is often coded language for marginalized Black communities who are forced to endure systemic racism leading to poverty and community violence as people struggle to survive. Rather than perception and opinion, evidence-based data does not support that “dangerous” neighborhoods or hospitals serving high numbers of Black patients are at increased risk of hospital shootings [2] or increased weapons confiscation [5].

Finally, authors also cite three studies by McNamara (1997), Mattox (2000), and Meyer (1997) to support the notion that the majority of surveyed patients, visitors, and staff favorably perceive the use of metal detectors. It is important to note that the mere perception of increased safety (or risk of violence) does not equate to an evidence-based reduction in incidences of workplace violence (or demonstrated need for policing). Additionally, those three studies are all over 20 years old and fail to prioritize medicine's ethical mandate to safeguard the physical and psychological safety of the most marginalized patients enduring well-known police brutality, like patients of color and patients with severe mental illness.

Concerningly high rates of hospital workplace violence undoubtedly require multifaceted approaches including quality security personnel and practices. However, researchers must guard against biased assumptions and flawed methodology. Though countercurrent to popular opinion and perception, evidence suggests that using funds towards improving clinical operations might reduce the multiple forms of healthcare violence better than funding metal detectors for thankfully rare armed hospital assaults.

## Declaration of competing interest

The authors declare that they have no known competing financial interests or personal relationships that could have appeared to influence the work reported in this paper.
